# Distribution of Copper, Iron, and Zinc in the Retina, Hippocampus, and Cortex of the Transgenic APP/PS1 Mouse Model of Alzheimer’s Disease

**DOI:** 10.3390/cells12081144

**Published:** 2023-04-13

**Authors:** Seyed Mostafa Hosseinpour Mashkani, David P. Bishop, Newsha Raoufi-Rad, Paul A. Adlard, Olga Shimoni, S. Mojtaba Golzan

**Affiliations:** 1 Institute for Biomedical Materials and Devices, School of Mathematical and Physical Sciences, Faculty of Science, University of Technology Sydney, 15 Broadway, Sydney, NSW 2007, Australia; 2 Hyphenated Mass Spectrometry Laboratory (HyMaS), School of Mathematical and Physical Sciences, Faculty of Science, University of Technology Sydney, 15 Broadway, Sydney, NSW 2007, Australia; 3 Synaptic Neurobiology Laboratory, The Florey Institute of Neuroscience and Mental Health, The University of Melbourne, Melbourne, VIC 3000, Australia; 4 Vision Science Group, Graduate School of Health (GSH), University of Technology Sydney, 15 Broadway, Sydney, NSW 2007, Australia

**Keywords:** Alzheimer’s disease, retina, transition metals, laser ablation inductively coupled plasma-mass spectrometry

## Abstract

A mis-metabolism of transition metals (i.e., copper, iron, and zinc) in the brain has been recognised as a precursor event for aggregation of Amyloid-β plaques, a pathological hallmark of Alzheimer’s disease (AD). However, imaging cerebral transition metals in vivo can be extremely challenging. As the retina is a known accessible extension of the central nervous system, we examined whether changes in the hippocampus and cortex metal load are also mirrored in the retina. Laser ablation inductively coupled plasma-mass spectrometry (LA-ICP-MS) was used to visualise and quantify the anatomical distribution and load of Cu, Fe, and Zn in the hippocampus, cortex, and retina of 9-month-old Amyloid Precursor Protein/Presenilin 1 (APP/PS1, *n* = 10) and Wild Type (WT, *n* = 10) mice. Our results show a similar metal load trend between the retina and the brain, with the WT mice displaying significantly higher concentrations of Cu, Fe, and Zn in the hippocampus (*p* < 0.05, *p* < 0.0001, *p* < 0.01), cortex (*p* < 0.05, *p* = 0.18, *p* < 0.0001) and the retina (*p* < 0.001, *p* = 0.01, *p* < 0.01) compared with the APP/PS1 mice. Our findings demonstrate that dysfunction of the cerebral transition metals in AD is also extended to the retina. This could lay the groundwork for future studies on the assessment of transition metal load in the retina in the context of early AD.

## 1. Introduction

Alzheimer’s disease (AD) is a common, incurable, and progressive dementia sub-type which is pathologically characterised by the formation of neurofibrillary tangles (NFTs) and senile plaques (SPs) through the hyperphosphorylation of tau protein and deposition of Amyloid-β (Aβ) protein [1]. There is extensive research evidence on the physiological distribution and homeostasis of transition metal ions such as iron, copper, and zinc, and in particular, their role in maintaining normal physiological functions in the brain, including signal transduction, energy production, and neurotransmitter synthesis [2]. Current evidence suggests that dyshomeostasis of transition metals, which leads to the formation of toxic oxidative species, has significant implications for the formation of amyloid plaques [3,4], the tau hyperphosphorylation process [3,5,6], and neuroinflammation associated with the pathological progression of AD [7,8,9]. The brain is vulnerable to free radical damage and oxidative stress since neuronal cell membranes consist of high levels of polyunsaturated lipids [10]. 

Transition metals have been identified as potential novel targets for therapeutic intervention [11]. High concentrations of Zn (1 mM), Cu (0.4 mM), and Fe (1 mM) have been reported within amyloid plaques [12] and previous studies have suggested that chelating Fe, Cu, and Zn from amyloid plaques can reduce their toxicity and consequently, increase their solubility, which further demonstrates the role of Fe, Cu, and Zn in AD pathophysiology [12,13,14]. 

To produce a profound understanding of how transition metals are implicated in neurodegenerative diseases such as Alzheimer’s and Parkinson’s disease, it is imperative to assess the metal concentration and distribution changes within regions affected by the disease process. A range of brain imaging modalities including computed tomography (CT) [15], magnetic resonance imaging (MRI) [16,17], and positron emission tomography (PET) [17] have been used to establish the presence of AD pathology. Nevertheless, they all suffer from common drawbacks, including high costs, limited accessibility, poor sensitivity, and specificity, [18] and the absence of standardization and scalability [19]. Typically, metal concentrations in anatomical regions have been measured through cutting, digestion, and analysis using various analytical techniques [20]. However, these techniques result in losing spatial information which is vital when the disease states being assessed involve small, well-defined regions or specific cell types. In light of this, laser ablation-inductively coupled plasma-mass spectrometry (LA-ICP-MS), as a means of ultra-sensitive chemical analysis with a part per billion detection limit, has received much attention for visualizing metals in biological systems, such as intact samples and tissue sections [21]. LA-ICP-MS has been applied for quantitative imaging of Cu, Fe, Zn, and Mn in the MPTP mouse model of Parkinson’s disease [22]; measuring the Mn, Fe, Cu, and Zn concentrations in the brain of a rat model of Parkinson’s disease [23]; copper mapping in a zebrafish model of Menkes disease [24]; imaging of Cu, Zn, Pb, and U in human brain tumour resections [25]; measuring Zn and Fe concentrations in the mouse model of traumatic brain injury (TBI) [26]; and imaging of iron in the frontal cortex [27], hippocampus [28], and white and grey matter [29] of healthy controls and Alzheimer’s disease patients.

The eye, and specifically the retina, is an extension of the central nervous system [30]. A growing body of evidence has linked retinal changes to pathophysiological features of AD, making the eye a strategic roadmap for screening and monitoring AD progression, particularly in its preclinical stages [19,31,32]. The retina is not restricted by some of the aforementioned limitations associated with brain imaging, and thus retinal imaging offers an attractive solution when studying AD-specific biomarkers. Whilst alterations in transition metals in the brain have been characterised before [33], little is known about whether such changes are also mirrored in the retina. Findings from studies that have linked retinal changes to pathological changes in the brain in AD [34] lead us to the hypothesise that a change in cerebral transition metal levels should be paralleled in the retina. Using LA-ICP-MS, we characterised the spatial distribution and quantified expression of Cu, Fe, and Zn in the hippocampus, the cortex, and the retina of 9-month-old Amyloid Precursor Protein/Presenilin 1 (APP/PS1) and Wild Type (WT) mice. The outcomes from this study may provide an insight in understanding transition metal alterations in the retina in AD. 

## 2. Materials and Methods 

All experiments were conducted within the Graduate School of Health (GSH) at the University of Technology, Sydney (UTS). APP/PS1 and normal aged mice were obtained from the Florey Institute of Neuroscience and Mental Health. All animal experimental procedures were approved by the Florey Institute of Neuroscience Animal Ethics Committee prior to the commencement of experiments (19-060-FINMH).

### 2.1. Animals

APP/PS1, a double transgenic mouse expressing a chimeric mouse/human amyloid precursor protein (Mo/HuAPP695swe) and a mutant human presenilin 1 (PS1-dE9) and age-matched C57BL6 WT mice were used in our experiments. We chose to examine animals at 9 months of age, as this reflects Aβ deposition in the hippocampus, cognitive impairment, and also impaired long-term potentiation (LTP) in the CA1 region of the hippocampus [35]. 

### 2.2. Tissue Collection

A total of twenty mice with an equal gender distribution were included in this study (10 APP/PS1 and 10 WT; 5 males and 5 females in each group). Following euthanasia with sodium pentobarbitone (80 mg/kg), transcardial perfusion was performed using 0.1 M phosphate buffer saline (PBS). The brain and whole eyes were removed, immediately placed in paraformaldehyde (4% *w*/*v*) and stored at 4 °C overnight. They were then cryopreserved in a 30% sucrose solution (PBS) for three days. Tissues were finally placed in an appropriate size mould and filled with optimal cutting temperature compound (OCT) and stored at −80 °C. Tissues were sectioned using the Leica CM1950 (Leica biosystem) at a thickness of 10 μm.

### 2.3. LA ICP-MS Imaging

LA ICP-MS was employed to measure the concentration of Cu, Fe, and Zn and their spatial distribution in the hippocampus, cortex, and retina of APP/PS1 and WT mice. The study was carried out on an Elemental Scientific Lasers NWR193 laser hyphenated to an Agilent Technologies 7700 ICP-MS, with 3 mL min^−1^ H_2_ added in the reaction cell [36] and argon used as the carrier gas. LA-ICP-MS conditions were optimized on NIST 612 Trace Element in Glass CRM. The samples were ablated under 50 µm spot size and a scan speed of 200 µm/s at a frequency of 20 Hz.

### 2.4. Image Analysis

The data were collated into a single image file using in-house developed software, Pew^2^, [37] and imported into ImageJ (National Institute of Health, MD, USA). The Allen Mouse Brain Atlas was used as reference [38] to draw a contour on the boundary of all regions of interest (hippocampus, cortex, and retina). DAPI staining was also used to visualize the gross anatomical morphology and to better guide the process of identifying region-specific anatomical boundaries. Following this process, the mean grey intensity value of each region was measured using the ImageJ built-in function. A minimum of 3 images per region were analysed and the average value taken as representative mean metal load.

### 2.5. Statistical Analysis

All statistical analysis was performed using Graphpad Prism (Dotmatics, CA, USA). Results are presented as mean ± standard error of the mean (SEM). Normality of data distribution was assessed using the D’Agostino and Pearson test. Unpaired *t*-test was used to compare differences between the two animal groups for each of the metals and anatomical regions. 

## 3. Results

### 3.1. Copper (Cu)

**Figure 1:**
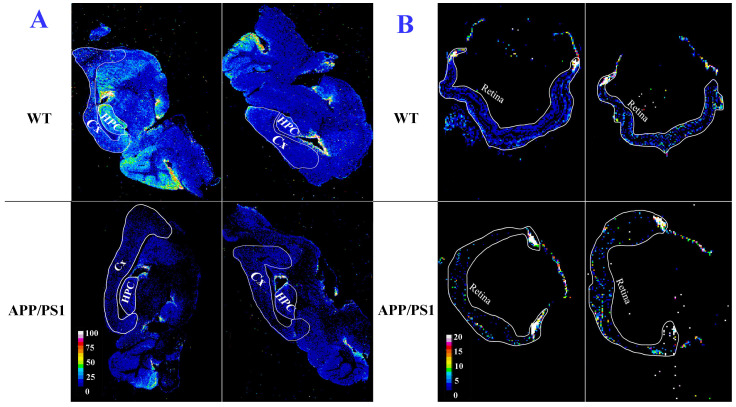
Spatial distribution of Cu in the brain (**A**) and eye (**B**). In each panel: sample map of 63Cu in sagittal brain/eye sections of 9-month-old WT (**upper row**) and APP/PS1 (**lower row**) mice. The scale represents calibrated Cu in ppm. HPC, hippocampus; CX, cortex. Side by side images of the brain and retina belong to two different animals.

**Figure 2:**
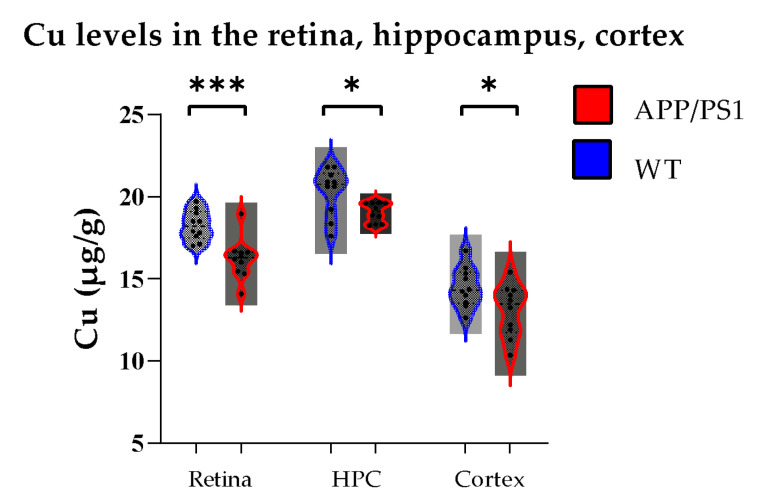
Quantified Cu levels. In all anatomical regions studied, Cu was significantly higher in WT (*n* = 10) compared with APP/PS1 (*n* = 10) mice. Error bars represent the Standard Error of the Mean (SEM). (* *p* < 0.05, *** *p* < 0.001).

The anatomical distribution of Cu in the brains and retinas is shown in Figure 1A,B, respectively. Visual assessment of the calibrated quantitative images demonstrated greater Cu enrichment in the hippocampus and cortex of WT mice compared with APP/PS1 mice (Figure 1A). A similar pattern was also observed in the eye, with the retina of WT mice harbouring higher concentrations of Cu compared to the APP/PS1 mice (Figure 1B). Results from the quantified image analysis were consistent with the visual assessment, with higher concentrations of Cu observed in the hippocampus, cortex, and retina of WT mice compared with APP/PS1 mice (Figure 2). More specifically, retinal Cu concentrations (µg/g) were significantly higher in WT mice compared with APP/PS1 mice (18.2 ± 0.9 vs. 16.2 ± 1.2, *p* < 0.001). In the hippocampus and cortex, Cu levels (µg/g) were also significantly higher in WT mice compared with APP/PS1 mice (20.3 ± 1.4 vs. 19 ± 0.5, *p* < 0.05 for the hippocampus; 14.4 ± 1.2 vs. 13 ± 1.5 *p* < 0.05 for the cortex).

### 3.2. Iron (Fe)

**Figure 3:**
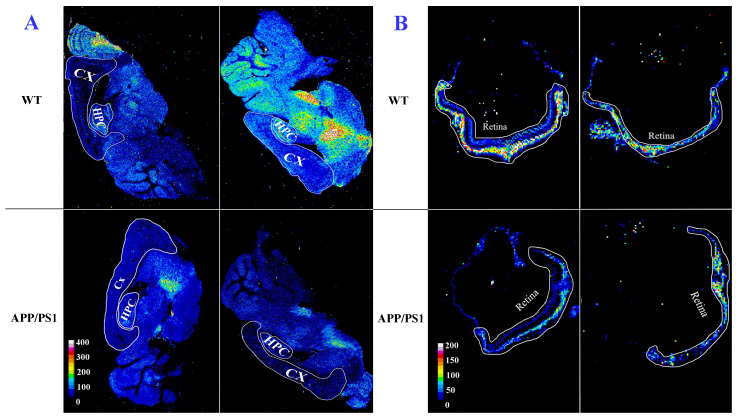
Spatial distribution of Fe in the brain (**A**) and eye (**B**). In each panel: sample map of 56Fe in sagittal brain/eye sections of 9-month-old WT (**upper row**) and APP/PS1 (**lower row**) mice. The scale represents calibrated Fe in ppm. HPC, hippocampus; CX, cortex. Side by side images of the brain and retina belong to two different animals.

**Figure 4:**
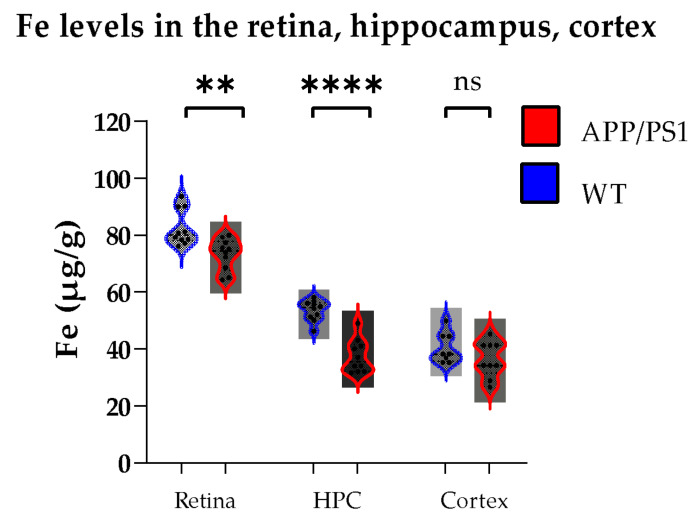
Quantified Fe levels. In the retina and hippocampus, Fe was significantly higher in WT (*n* = 10) compared with APP/PS1 (*n* = 10) mice. The Fe load in the cortex was not significantly different between the two groups (*p* = 0.18). Error bars represent the Standard Error of the Mean (SEM). (** *p* < 0.01, **** *p* < 0.001).

The spatial distribution of Fe in the brain and retinal sections of a representative WT and APP/PS1 mouse is shown in Figure 3A,B. Visual assessment of the calibrated quantitative images of the brain shows that the hippocampus and cortex of WT mice possess higher concentrations of Fe compared with APP/PS1 mice (Figure 3A). The retina of WT mice displayed a similar pattern (higher Fe content) relative to the age-matched APP/PS1 mice (Figure 3B). The intensity-based image analysis also confirmed these observations: the hippocampus, the cortex, and the retina of WT mice have higher Fe concentrations than APP/PS1 mice (Figure 4). In the retina and hippocampus, Fe concentrations (µg/g) were significantly higher in WT mice compared with APP/PS1 mice (82.5 ± 6.3 vs. 73.1 ± 5.5, *p* < 0.01 for retina, 53.5 ± 3.6 vs. 37.3 ± 5.7, *p* < 0.0001 for hippocampus). In the cortex, while Fe concentration (µg/g) in the WT mice was higher than that of APP/PS1 mice, the difference was not statistically significant (40.4 ± 5.3 vs. 36.4 ± 6.3, *p* = 0.18).

### 3.3. Zinc (Zn)

**Figure 5:**
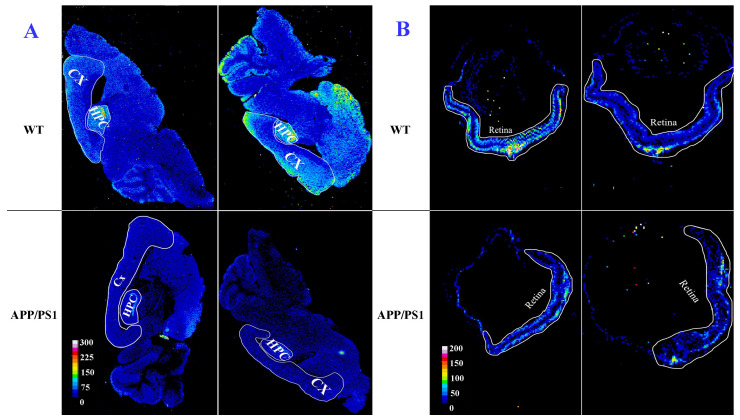
Spatial distribution of Zn in the brain (**A**) and eye (**B**). In each panel: sample map of 66Zn in sagittal brain/eye sections of 9-month-old WT (**upper row**) and APP/PS1 (**lower row**) mice. The scale represents calibrated Zn in ppm. HPC, hippocampus; CX, cortex. Side by side images of the brain and retina belong to two different animals.

**Figure 6:**
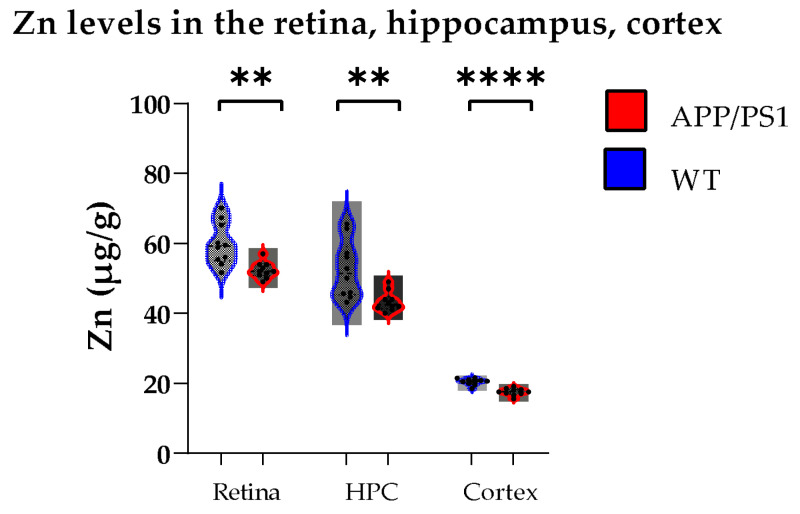
Quantified Zn levels. In all anatomical regions studied, Zn was significantly higher in WT (*n* = 10) compared with APP/PS1 (*n* = 10) mice. Error bars represent the Standard Error of the Mean (SEM). (** *p* < 0.01, **** *p* < 0.0001).

The quantitative images of Zn distribution in the brains and eyes are shown in Figure 5A,B. Surveying the calibrated quantitative images, they illustrate higher Zn concentration in the hippocampus and cortex of WT mice compared with APP/PS1 mice (Figure 5A). Similarly, the retina of WT mice also displayed the same trend, containing higher zinc levels than the APP/PS1 mice (Figure 5B). The intensity-based image analysis demonstrated significantly higher concentrations of zinc (µg/g) in the hippocampus, cortex, and retina of WT mice compared with APP/PS1 mice (59.8 ± 6 vs. 52.3 ± 2.2, *p* < 0.01 for the retina; 52.5 ± 8 vs. 43.3 ± 2.7, *p* < 0.01 for the hippocampus; 20.4 ± 0.9 vs. 17.5 ± 1, *p* < 0.0001 for the cortex) (Figure 6).

## 4. Discussion

In this study, we used LA-ICP-MS to study the spatial distribution of Cu, Fe, and Zn in cross-sectional slices obtained from the brains and the eyes of 9-month-old APP/PS1 and WT mice. Calibrated image-based intensity analysis was also employed to quantify transition metal levels in each of these structures. Our findings showed that the retina, hippocampus, and cortex of WT samples possessed higher Cu, Fe, and Zn levels than APP/PS1 mice. Collectively, our findings suggest that metal dyshomeostasis observed in the hippocampus and cortex are also mirrored in the retina.

Various analytical techniques, such as secondary ion mass spectrometry (SIMS) [39], particle-induced X-ray emission (PIXE) [40], X-ray fluorescence (SXRF) microprobe [41], graphite furnace atomic absorption spectrometry (GFAAS) [42], neutron activation analysis (NAA) [43], and instrumental neutron activation analysis (INAA) [44] have been used for bio-elemental dissection of AD pathology. Using PIXE and SXRF, altered levels of iron and zinc has been reported in the hippocampi of human AD brains as well as PSAPP mice [40,45]. LA-ICP-MS, as the biological trace element imaging technique, has advantages over the techniques mentioned above, such as high sample output, high sensitivity, high accuracy and precision of the analytical data, no charging-up effects, and fewer matrix effects, which results in a simple quantification of analytical data at a fraction of the cost of other techniques. These advantages motivated us to employ LA-ICP-MS for visualizing and quantifying Cu, Fe, and Zn in the retina, hippocampus, and cortex of APP/PS1 mice. 

### 4.1. Copper

Copper is a necessary element for maintaining healthy cellular processes in the brain and the eye. In the brain, copper is vital for metalloenzyme functions which are essential in metabolic processes such as energy metabolism, antioxidant-defence mechanisms, and neurotransmitter synthesis [46,47]. In the retina, and similarly in the brain, “free” Cu participates in neurotransmission [48]. While copper overload is toxic, copper deficiency results in morphological changes in retinal structures [49]. The quantified total copper in the human retina has been reported as 6.6 ± 1.4 and 9.0 ± 5.0 μg/g in the RPE/choroid and neuroretina, respectively [50].

An age-associated increase in Cu levels has been reported in the brains and retinas of humans and mice [50,51], and in AD, a mis-metabolism of Cu has been associated with oxidative stress [52]. Copper has an impact on the regulation of other transition metals in AD [53]. Aβ precursor protein (APP) molecules are cleaved into two pathways (non-amyloidogenic and amyloidogenic) in the presence or absence of Cu [54]. The amyloidogenic pathway leads to amyloid generation. Previous studies have reported that Cu^+2^ ions prevent amyloid formation by reacting with a γ-secretase complex [4] or disturbing APP dimerization [55]. Therefore, low Cu concentration in AD raises Aβ production and accumulation by enhancing the amyloidogenic processing of APP [56]. 

Cu ions [57], through reduction from Cu^2+^ to Cu^1+^, play a protective role against free radicals, an underlying cause of mitochondrial oxidative damage. Lower Cu content leads to higher levels of free radicals, as observed in amyloid plaques [58]. This could explain our observations of significantly lower Cu levels in the APP/PS1 mouse model of AD compared with WT mice. However, further studies are required to determine how Cu metabolism is disrupted in the brain of APP/PS1 mice and whether a similar process is also responsible for retinal Cu deficiency observed in these animals.

### 4.2. Iron

Iron is involved in a variety of essential metabolic processes, including oxygen transport, electron transport, DNA synthesis, redox/non-redox reactions, and other cell functions [59]. Iron is a highly redox-active compound, and due to its role in the generation of ROS, it is tightly regulated [60]. Changes in cerebral Fe levels have been proposed as a marker for cognitive impairment in AD [61]. Iron dyshomeostasis can instigate Aβ creation and aggregation because Fe is able to act on the iron regulatory element (IRE) site of APP mRNA, consequently boosting the APP translation and expression [62]. Furthermore, iron can increase the beta-secretase cleavage of APP, which enhances Aβ production by preventing alpha-secretase-induced APP cleavage via furin [63]. Moreover, iron can bind to the tau protein and boost the tau phosphorylation process, resulting in increasing hyperphosphorylated tau aggregation into neurofibrillary tangles. 

Iron is also critical for maintaining retinal function [64], with retinal iron dyshomeostasis resulting in ocular diseases, such as glaucoma, cataract, AMD, and conditions causing intraocular haemorrhage [65]. It has been reported that the blood-retinal barrier protects the retina from systemic iron loading [66]. However, higher intraocular iron concentration leads to oxidative damage to the retina, which results in lipid peroxidation of photoreceptors and retinal degeneration [67]. Iron is mainly present in the choroid, the retinal pigment epithelium (RPE), and photoreceptors layer [68,69]. Using Graphite furnace atomic absorption spectrometry (GFAAS) and inductively coupled plasma mass spectrometry (ICP-MS) techniques [70], a mean Fe load of 117.63 ± 14.58 μg/g has been reported in the human neuroretina [71] 

Many studies have reported an overload of Fe in AD, which contributes to Aβ deposition [72,73,74,75]. Despite this, we observed a uniform Fe deficiency in the retina, hippocampus, and cortex of APP/PS1 mice compared to their counterpart WT animals. This could be explained using a study by Maynard et al. [51] that suggested high levels of the carboxyl-terminal fragment of APP in the AD mouse brain can lead to a reduction in Fe levels [51]. In addition, the lower level of iron regulatory proteins, including hepcidin, iron-homeostatic peptide, and ferroportin, in AD results in lower Fe levels [76]. A study using APP/PS1 mice [77], the same model used in our study, has established that Fe is chemically reduced in the presence of aggregating Aβ plaques under certain physiological conditions. This could also explain the reduction in retinal Fe levels, as Aβ plaques have also been reported in the retina in AD [31]. Nevertheless, further mechanistic studies are required to interrogate this theory. 

### 4.3. Zinc

Zinc, the second most abundant micronutrient in the human body, is a significant player in biological processes such as maintaining protein structure and stability, enzyme activities, adjusting various cellular processes, signal transduction, learning, and memory, and in the development and integrity of the immune system [78,79]. In the brain, Zn^2+^ is synaptically diffused during neuronal activity and plays a vital role in axonal and synaptic transmission and is obligatory for nucleic acid metabolism and brain tubulin growth and phosphorylation [80]. High levels of Zn are also present in the retina, suggesting a critical role in retinal physiology [81]. ICP-MS study of retinal pigment epithelium (RPE)/choroid and neuroretina demonstrated a mean Zn value of 292.1 ± 98.5 and 123.1 ± 62.2 μg/g, respectively [82]. Zn deficiency leads to ultrastructural changes in the retina and retinal pigment epithelium [83]. 

A high concentration of Zn^2+^ ions has been found in senile plaques of post-mortem AD brains [12,84]. Zinc ions interact with histidine residues at the N-terminal of Aβ [85,86]. However, the Aβ aggregation mechanism of Zn^2+^ is still elusive because assemblies of Zn-Aβ are variable and sensitive due to changes in pH, temperature, concentration, and buffer environment [87]. Accordingly, elevated levels of zinc have been reported in AD [88]. Interestingly, our results demonstrate lower Zn levels in the retina, hippocampus, and cortex of APP/PS1 compared with WT mice. A likely explanation for our finding is based on the fact that previous studies have shown that Zinc Transporter 3 (ZnT3) proteins are essential for loading Zn into synaptic vessels [89,90,91]. These proteins are abundantly available during the early stages of AD and its only with ageing that ZnT3 levels are depleted resulting in higher levels of free Zn. We did not evaluate ZnT3 expression in our study, as it fell outside the scope of the project, and therefore cannot determine whether an alteration in ZnT3 levels is driving the changes seen in Zn levels in our animals. A similar event could also be extended to the retina, as ZnT3 has been localised to the neural retina in regions which have been found reactive for Zn ions [92]. Zn suppression in senile plaques could also contribute to the smaller pool of synaptic zinc observed [93]. Further work is required to examine how Zn and its transporters are altered in the brain and the retina. 

### 4.4. Limitations

Despite the current study being the first to visualise and objectively quantify transition metal levels in the retinas and brains of APP/PS1 and WT mice, our study has several limitations. First, as distribution of iron, zinc, and copper in the retina could be non-uniform, our approach of evaluating metal load in the retina as a whole may encompass biological bias. Second, we only studied animals at 9 months of age, which may have not captured the full continuum of age-associated changes in metal dyshomeostasis over the course of the disease. Third, transgenic animal models are artificially modified at a genetic level; hence, they may not represent the complex multifactorial origin of the most common form of AD, the sporadic variant [94]. AD progression in transgenic animal models happens in a very different time window than in AD patients [95]. As our study did not include a negative control group, it could be possible that the changes observed in our study may be a result of the genetic manipulation in our animal cohort. Whilst the distribution and metal load observed in our WT mice is broadly comparable to a study that used LA-ICP-MS to quantify metal load in 4–5 month-old C57BL/6 mice [96], further experiments using our findings are required. Future studies should also include analysis of the individual retinal layers as well as studying animals at various ages on the disease spectrum. 

### 4.5. Conclusions

Whilst previous studies have used LA-ICP-MS to quantitatively image transition metals in the brain of AD mouse models as well as post-mortem donor human tissue samples [22,27,28], our study was the first to visualize and quantify the spatial distribution of Cu, Fe, and Zn in the retina in addition to the brain, and more specifically the hippocampus and the cortex of an AD mouse model. Our findings showed that the hippocampus, cortex, and retina of WT samples express significantly higher Cu, Fe, and Zn than APP/PS1 mice. Overall, our results suggest that an imbalance in retinal transition metal levels occurs in parallel in the hippocampus and cortex in AD, and this lays the groundwork for further work studying retinal metal load in the context of early onset Alzheimer’s disease.

## Data Availability

The data presented in this study are available on request from the corresponding author.
